# Research Advances on Stem Cell-Derived Extracellular Vesicles Promoting the Reconstruction of Alveolar Bone through RANKL/RANK/OPG Pathway

**DOI:** 10.3390/jfb14040193

**Published:** 2023-03-30

**Authors:** Xia Huang, Yuxiao Li, Hui Liao, Xin Luo, Yueping Zhao, Yadong Huang, Zhiying Zhou, Qi Xiang

**Affiliations:** 1 Institute of Biomedicine and Guangdong Provincial Key Laboratory of Bioengineering Medicine, Jinan University, Guangzhou 510632, China; 2 School of Stomatology, Jinan University, Guangzhou 510632, China; 3 Department of Orthodontics, The First Affiliated Hospital of Jinan University, Guangzhou 510632, China

**Keywords:** stem cell-derived extracellular vesicles, reconstruction of alveolar bone, osteogenesis, RANKL/RANK/OPG pathway

## Abstract

Periodontal bone tissue defects and bone shortages are the most familiar and troublesome clinical problems in the oral cavity. Stem cell-derived extracellular vesicles (SC-EVs) have biological properties similar to their sources, and they could be a promising acellular therapy to assist with periodontal osteogenesis. In the course of alveolar bone remodeling, the RANKL/RANK/OPG signaling pathway is an important pathway involved in bone metabolism. This article summarizes the experimental studies of SC-EVs applied for the therapy of periodontal osteogenesis recently and explores the role of the RANKL/RANK/OPG pathway in their mechanism of action. Their unique patterns will open a new field of vision for people, and they will help to advance a possible future clinical treatment.

## 1. Introduction

The periodontium plays an important role in people’s daily lives. The entire periodontal structure consists of the gingiva exposed to the oral cavity, the alveolar bone covered by the gingiva, and the cementum covering the roots of the teeth. Periodontal ligaments are created when Sharpey’s fibers enter the cementum-connected alveolar bone. One of the most infamous inflammatory diseases is periodontal disease, which starts as an external inflammatory reaction of the gingiva (gingivitis) and later proceeds to clinical attachment loss with the degeneration of the inflammatory in periodontal structure (periodontitis). The height of the alveolar ridge decreasing and bone loss are the disease’s major defining characteristics [1]. Furthermore, external apical root resorption (EARR), a pathological side effect of orthodontic treatment, also causes bone loss and the reconstruction of the alveolar bone [2]. All of these conditions share the trait that the more advanced the progression, the more challenging they become to solve, as well as for regeneration to occur.

In the past few decades, people have explored bone regeneration treatments for alveolar bone defects. Currently, the main methods used to treat this problem are autologous or allogeneic bone transplantation and guided tissue regeneration (GTR). However, autologous bone grafts cannot be used for repairs in patients with large defects. Allogeneic bone fillings are accompanied by complications, such as bone defects and infections. GTR involves the use of absorbable collagen membranes to cover the defect area of alveolar bone, with the goal of achieving alveolar bone regeneration [3]. However, GTR treatments are not stable, owing to their multiple types of complications. Periodontal osteogenesis has received attention, as it relates to the treatment of common oral disorders, such as periodontal treatment, dental implants, orthodontic tooth movement, and disease recurrence. Therefore, we urgently need to find a more feasible way to promote treatments for periodontal osteogenesis.

Once, stem cell therapy was considered to be the most promising strategy for treating periodontal bone loss. In stem cell treatments, autologous or allogeneic stem cells are isolated and amplified in vitro and then planted on a natural or synthetic cell scaffold with a good biocompatibility, where they undergo gradual degradation until being absorbed by human body. This biomaterial scaffold provides a three-dimensional (3D) space for the cells to survive in, and the stem cells grow on the three-dimensional scaffold prefabricated before. Then, the hybrid material is implanted into periodontal defects to form alveolar bone-like tissues [4]. However, there are many problems with this clinical technique that have yet to be resolved, such as the possibility of autoimmune cell rejection, the high cost and technical difficulty, and the issue of stem cells holding low survival rate in stem-cell therapy, and the risk of cancer or tissue disease should also be taken in consideration [5]. Later, a large number experiments confirmed that extracellular vesicles derived from various stem cells (SC-EVs) have the same therapeutic efficacy or even more advantages than the original stem cells for tissue repair [6,7]. SC-EVs are nanosized membrane particles secreted by stem cells, such as pulp stem cells, bone marrow stem cells, and human umbilical cord mesenchymal stem cells, which carry diverse biological information, including DNA, RNA, bioactive lipids, proteins, and other biologically related molecules [8]. The use of SC-EV-assisted osteogenesis to treat periodontal bone loss [9,10,11] vastly withstands the risk of self-rejection and improves the safety and stability of treatments. It is noteworthy that stem cell therapy presents the common challenge of inability to resist cytotoxicity, either biobanking of the useful secretome at the inflammatory region [12].

Amid the course of alveolar bone remodeling, the RANKL (RANK ligand)/RANK (receptor activator of NF-κB)/OPG (osteoprotegerin) signal pathway is involved in bone resorption through its main work in osteoclast separation and enactment, as well as within the provocative reaction [6]. Trabecular bone resorption by osteoclasts is a necessary regulated process that frees an area for osteoblasts to form new bone [13]. Especially in orthodontic practice, the success of tooth movement is reliant on the RANKL/RANK/OPG signaling pathway, which applies pro-osteogenic and anti-inflammatory impacts within the handle of remaking of the alveolar bone.

An experimental study on the use of SC-EVs in periodontal osteogenesis, which explored the relationship between SC-EVs and the RANKL/RANK/OPG pathway, found that this signaling balance is an important contributing factor to bone remodeling using SC-EVs. The status of these signaling pathway axis is the key for the success of orthodontic and implant oral therapy. Further research progress on the use of SC-EVs for the treatment of alveolar bone osteogenesis via the RANKL/RANK/OPG pathway will help with the reconstruction of alveolar bone and the fabrication of more suitable and biocompatible scaffold materials based on SC-EVs. There are fewer antigens on the surface of exocrine membranes, which reduces immune rejection while retaining the bioactive components of stem cells. Therefore, we think the application of SC-EVs will have the best osteo-inductive properties and become a compelling new form of “stem cell-free” therapy.

## 2. The Reconstruction of Alveolar Bone

Alveolar bone remaking may be a persistent and slow preparation. It depends on the energetic adjust between bone retention and bone arrangement. At first, osteo-clast-mediated bone retention is actuated, and after that, unused osteoblast-mediated bone arrangement starts [14].

Bone retention and bone formation are interconnected, and they depend on the reg-ulation of each jolt figure so that modern bone can supplant ancient bone and stabilize the volume of alveolar bone. Nearby components incorporate microdamage and aggravation, which initiate osteocytes to deliver variables that invigorate bone resorption. Specifically, the stimulus factors include: (i) biochemical factors established by a variation in hormone secretion, including the parathyroid hormone (PTH) [15] and estrogen [16]; (ii) an increase in some chemokines [17] and cytokines [18], which can indirectly promote osteoclastogenesis; and (iii) mechanical components that are set up by the mechanical stacking connected to bone [19,20]. Normally, an extracellular signal passes through the cell membrane, enters into the cell, and is converted into a chemical signal. Then, the chemical signal is transferred into the cell to activate osteoclast differentiation, proliferation, migration, and processes. However, in periodontitis, this balance is destroyed. Periodontitis mainly refers to pathological alveolar bone resorption and partial new bone formation. In the mechanism of periodontitis-related bone loss, virulent periodontal pathogens overwhelm the defense barrier of the oral mucosa. In response, the resident cells in the tissues, such as fibroblasts, keratinocytes, and dendritic cells, discharge incendiary cytokines, advance different incendiary cells to deliver anti-bacterial specialists, and create re-active oxygen species to dispose of the pathogens, but this, moreover, crushes the typical activities of alveolar bone remodeling [21]. Osteoclasts are initiated and enacted, whereas osteoblasts are repressed, which can crush the bone adjust and lead to a diminishment in bone volume.

Orthodontic tooth movement (OTM), moreover, involves alveolar bone reconstruction. OTM basically alludes to physical alveolar bone resorption and reconstruction. Amid OTM, bone resorption happens on the compression side of the alveolar bone, and osteogenesis happens on the pressure side of the alveolar bone. The orthodontic constraint exasperates the steady environment of the periodontal tendon space by changing the blood stream and nearby environment, causing a disturbance within the support of the alveolar bone status [22]. Orthodontic forces increase IL-1β and TNF-α in periodontal ligaments and induce PGE2 to be released from periodontal ligament cells and osteoblasts, which is also one of the important chemical mediators of compression-induced alveolar bone resorption [23]. These factors produced by the periodontal ligament cells on the compression side help RANKL promote bone resorption and experimental tooth movement. In contrast, tensile forces increase the osteogenic factors (e.g., TGF-β and IL-10) in periodontal ligaments, which may increase OPG and reduce RANKL production to inhibit osteoclast bone resorption.

In clinical cases, many patients have their damaged teeth extracted, but they cannot receive implant treatment precisely because the reconstruction of their alveolar bone is unsuccessful, such that mechanical stress on the implant teeth would have a negative impact on the insufficient bone tissue [24]. It is important to design an appropriate implant surface and alveolar bone regeneration materials in order to remove the osteoclasts and induce an inflammatory reaction to the implant, which is helpful to the alveolar bone. Many studies have focused on evaluating the effect of proteins coated on the surface of titanium implants and the effect of changing the surface roughness of implants on reducing osteoclasts. It has been found that coating the surface of inserts with a moo dosage of bone morpho-genetic proteins (BMPs) or OPG can improve bone arrangement and move forward bone integration [25].

**Figure 1:**
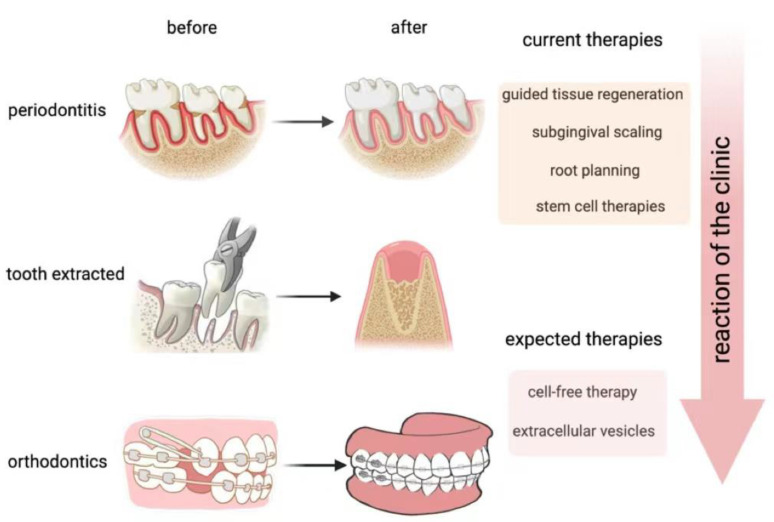
Development of methods for alveolar bone reconstruction. Above are typical scenes in clinical treatments, all of which are involved in alveolar bone reconstruction. We expect to achieve cell therapy by using extracellular vesicles to replace current therapies, such as guided tissue regeneration (GTR). (This figure was drawn using BioRender.com).

Thus, we aimed at reconstructing alveolar bone with appropriate morphological characteristics. As for periodontitis, we expected to increase the bone formation and slow down the bone resorption rate, thus regaining the balance between them, and even resulting in reverse pathological processes in order to restore the alveolar bone (Figure 1).

At present, dentists tend to cure periodontitis by using GTR, subgingival debridement, subgingival scaling, root planning, etc. [26]. These treatments can effectively stop the inflammation, but they can do nothing to restore alveolar bone formation. As for OTM, if the mechanical force is not properly balanced, it can lead to a lack of bone coverage at the root of the tooth, which can then result in the reconstruction period of alveolar bone being too long to allow for correct remodeling. Therefore, it is an urgent problem for orthodontic treatment to accelerate bone reconstruction under conditions that allow for better bone repair.

## 3. SC-EVs and Their Biological Characteristics

EVs are macromolecule lipid bilayer layer structure vesicles that are emitted by the larger part cells and are vital carriers of cell-to-cell communication. EVs carry a spread of biomolecules and speak to a substitution kind of data exchange, with subsequent proficiency and better specificity than flag transduction within the conventional sense. Moreover, EVs act as aggregates of information, extending the signal transduction to the intercellular level [27].

EVs are widely present in numerous body fluids, together with heterogeneous groups of membrane vesicles from various sources. They can carry a variety of biomolecules, including DNA fragments, RNA (including miRNA, mRNA, tRNA, and lncRNA), lipids, proteins, peptides, sugars, and metabolites [28]. Differences in the composition of the biomolecules determine the specificity of the EVs’ function. Reckoning on the kind of cell from that it comes, an EV can reveal specific proteins for that cell [29].

Based on EVs biogenesis and size, EVs are typically divided into three classes: exosomes (Exos), microvesicles (MVs), and apoptotic bodies. Exosomes, that are 50–150 nm, are intraluminal vesicles fashioned by the endosomal membrane budding internally all through the development of multivesicular bodies (MVBs), and MVBs are secreted when fusion occurs with the cytomembrane [30]. MVs are 100–1000 nm in diameter and are formed by the plasma membrane budding outward. They are released directly into the extracellular matrix [31]. The diameter of apoptotic bodies is usually defined as 1–5 μm, and apoptotic bodies form following cytoplasmic membrane blebbing in cells undergo programmed cell death, or apoptosis [32].

All EVs are formed by a phospholipid bilayer and carry important biological information from related cells. After being released from cells, EVs can realize biomolecular transformation by recognizing specific proteins on the surface of the recipient cells or entering the recipient cells to perform functions, including repairing tissue damage and promoting angiogenesis. EVs are seen as a promising delivery system that can send signals into cells through different mechanisms. Instead of delivering cargo, EVs interact with the molecules on the surface of the cell membrane to trigger cascades of reactions within the cell [33].

The procedures regularly utilized for exosome partition basically contain ultra-centrifugation, ultrafiltration, immunoaffinity capture, charge neutralization-based polymer precipitation, size-exclusion chromatography, and microfluidic procedures [34]. Be that as it may, these ordinarily utilized separation strategies possess benefits and disadvantages. Ultra-centrifugation is the gold standard of exosome isolation approaches, and it promptly translates to exosome isolation [35]. In recent years, the wide application of microfluidic technology has provided a replacement plan for the isolation of exosomes [36], supporting variations in their biological and physical properties, which permit for a highly sensitive and high-speed separation; but, the yield is low, and this is often solely appropriate for diagnoses. Additionally, more and more industrial kits are developed for the isolation of exosomes, which are simple and convenient to operate without special equipment, and with the upgrading of products, the extraction potency and purification of exosomes has improved in a gradual way.

**Figure 2:**
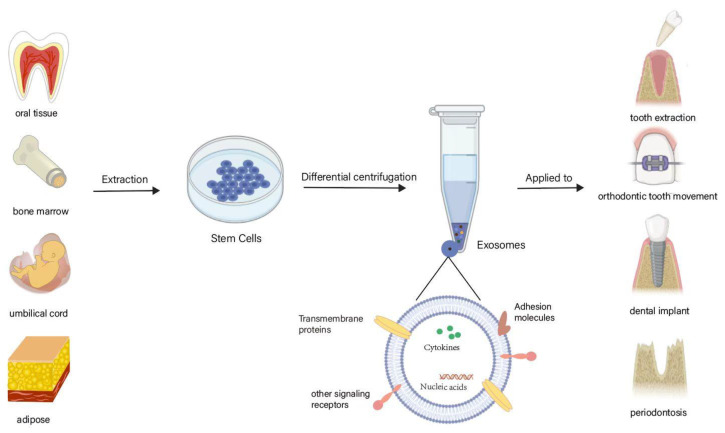
Use of SC-EVs within the field of oral medicine. Exos derived from some common stem cells act on teeth, as well as on the whole body. Of the stem cells derived from various tissues, oral stem cells are the most advantageous in terms of ethics and the level of difficulty in obtaining them. (This figure was drawn using BioRender.com and Adobe Illustrator 2021).

Stem cells are a category of undifferentiated pluripotent cells with broad prospects in regenerative medication thanks to their high self-renewal and multidirectional differentiation capabilities. EVs secreted by stem cells can show comparable capacities to stem cells, such as promoting tissue repair and regeneration [37]. Some examples of common mesenchymal stem cells (MSCs) include bone marrow MSCs (BMSCs), umbilical cord MSCs (UCMSCs), adipose-derived stem cells (ADSCs), and human periodontal ligament stem cells (hPDLSCs) (Figure 2).

BMSC-EVs have been shown to have immunomodulating, proliferative, tissue repair, anti-inflammatory, and antioxidant effects; in addition, they can regulate osteoclast production and osteogenic differentiation [38]. ADSC-EVs are primarily associated with graft rejection, skin healing, and apoptosis [39]. UCMSC-EVs play a major role in promoting neovascularization, inflammatory responses, and nerve regeneration [40].

With the gradual deepening of EV research, it has been found that SC-EVs can not only effectively transmit the signals of maternal stem cell-induced regeneration, showing the same therapeutic effect, but they can also lower the risk of self-rejection and enhance the safety of treatments [6]. When attacked by inflammation, the body will produce an immune response. A damaging immune response is not only detrimental to the damage repair of defects, but it also aggravates the occurrence of damage. SC-EVs can inhibit the immune response’s effect on damaged tissues [41]. In stem cell therapy, we use the pluridirectional differentiation potential of stem cells to realize the repair of tissue defects. Therefore, stemness is the most important aspect of stem cell-related therapy. Studies have shown that EVs extracted from embryonic stem cells (ESCs) can maintain the stemness of ESCs by activating the FAK pathway [42]. Thus, we suspect that SC-EVs can maintain the stemness of their original stem cells, such as human periodontal stem cells, which are often used to repair bone defects caused by periodontitis.

SC-EVs also have significant advantages in long-term stability. EVs or SC-EVs are usually frozen in PBS or normal saline, or in a specific cell-freezing medium, and they can remain stable during cryopreservation [43].

## 4. SC-EVs Promote Bone Tissue Repair and Regeneration

### 4.1. SC-EVs in Bone Tissue Repair and Regeneration

Due to SC-EVs having a wide range of sources and powerful biological functions, they are also widely used in tissue repair and regeneration, as well as biomarkers for disease diagnoses and other fields, and they have been verified in bone, heart, and other tissues. More importantly, SC-EVs are hydrophilic, which allows them to be loaded directly into soluble biomaterials, such as hydrogels. Electrostatic interactions also contribute to the ability of biomaterials to carry SC-EVs; for example, a positively charged biological scaffold can absorb negatively charged SC-Evs [44]. The bioactive adhesion of SC-Evs is beneficial because it allows the SC-Evs to adhere to the surface of different kinds of biological scaffolds, such as bioactive glass, bone matrix scaffolds, etc. [45].

Swanson et al. [10] extracted Exos from human dental pulp stem cells (hDPSCs), combined the Exos into nanofiber tissue-engineering scaffolds, and they implanted them into the skull defects of mice to examine the level of bone repair. This platform has great characteristics, such as huge surface range and huge porosity, permitting it to be embedded into Exos from three measurements, making it a perfect choice for functionalizing its surface by conveyance [46]. Then, a post-seeding technique was used to immobilize hDPSC-Exos on nanofibrous. The results of micro-CT scanning of the bone defect site showed that the bone density of the defect site increased significantly in the Exo stent group. Chitosan is a common medical biogel and a reliable controlled-drug-release material. Combined with EVs, chitosan based hydrogels promoted cell migration, angiogenesis, and re-epithelization [47]. When hDPSC-Exos are loaded into a chitosan hydrogel, DPSC-Exos can also play the role of osteosynthesis [48]. Diomede et al. [11] combined hPDLSC-derived EVs into a collagen membrane for skull defects in mice and found that, both in vitro and in vivo, EVs exhibited good osteogenic properties. hPDLSC-EVs effectively inherited the biological functions of hPDLSCs for osteogenic differentiation and the secretion of stromal cell-derived factor 1 (SDF1). As a result, the ponder effectively created an unused biocompatible osteogenic development, comprising a commercially accessible collagen film and hPDLSC-EVs or poly-ethylenimine (PEI)-engineered EVs (PEI-EVs). SC-EVs inferred from human umbilical line mesenchymal stem cells (hUCMSCs) and bone marrow-derived stem cells (BMSCs) can moreover proficiently express osteogenic markers [49]. Yang et al. [50] effectively repaired bone defects in mice by integrating Exos derived from hUCMSCs with hydrogels. Ideal vectors for EVs also include bone repair scaffolds with a stereoscopic structure, such as tricalcium β-phosphate (β-TCP) [51] and mesoporous bioactive glass (MBG) [52], which have more advantages in terms of adaptability to the EV size and the induction of osteogenesis [53]. Furthermore, these natural bone mimics are often biodegradable and exhibit a good biocompatibility. Recently, scientists developed a novel strategy to avoid the clearing of nanoparticle-fused EVs by the reticuloendothelial system (RES)/the mononuclear phagocytic system (MPS). The strategy involves saturating the MPS with macrophage-targeting EVs, and then engineering homing peptides to enhance the tissue-targeting features of EVs, which resulted in better therapeutic effects and the avoidance of side effects in the liver and spleen. This points out that safely and effectively generating bone-targeting EVs with low clearance rates is a future research direction [54,55].

### 4.2. SC-EVs in Alveolar Bone Tissue Repair and Regeneration

SC-EVs have been proven to have anti-inflammatory and immunosuppressive effects in different tissues. In addition, SC-EVs have strong bone tissue regeneration characteristics, and their safety level is high, which is necessary for a material for the regenerative treatment of alveolar bone loss.

Lei et al. [56] extracted small extracellular vesicles (sEVs) derived from PDLSCs (PDLSC-sEVs) from healthy periodontal subjects, and they used them in combination with a Matrigel hydrogel to repair bone defects in rat periodontitis models. They found that PDLSC-sEVs seem to save the osteogenic capacity of endogenous stem cells in inflammatory environments and contribute to alveolar bone regeneration. Watanabe et al. [57] studied the preventive and therapeutic effects of BMSC-EVs on bisphosphonate-associated jaw necrosis after a tooth extraction. The results stated that BMSC-EVs could reduce the number of senescent cells and inflammatory cytokines, increase the expression of the stem cell markers Bmi1 and Hmga2 and the vascular endothelial growth factor, prevent stem cells, osteoblasts, and fibroblasts from aging and the spread of chronic inflammation, promote the healing of the alveolar fossa, and effectively prevent the occurrence and development of bisphosphonate-related jaw necrosis. Nakao et al. [58] injected Exos derived from gingival mesenchymal stem cells (GMSC-Exos) and preconditioned with TNF-α (GMSC-Exo-TNF-α) into a ligation-induced periodontitis model in mice, and the effect of the treatment on inflammatory bone loss was observed. The results showed that a local injection of GMSC-Exos could lessen periodontal bone damage and decrease the amount of tartrate-resistant acid phosphatase(TRAP)-positive osteoclasts. GMSC-Exo-TNF-α further enhanced this effect, indicating that preconditioning with TNF-α was beneficial for regulating inflammation and osteoclast formation. A miRNA array analysis showed that TNF-α significantly stimulated the up-regulation of miR-1260b. miR-1260b can target Wnt5a and JNK1, which relate to the regulation of osteoclasts [59]. The above results show that SC-EVs are effective and practical non cellular therapies for periodontitis, and they can improve alveolar bone regeneration of periodontal support tissue.

## 5. RANKL/RANK/OPG Signaling Pathway and Alveolar Bone Osteogenesis

**Figure 3:**
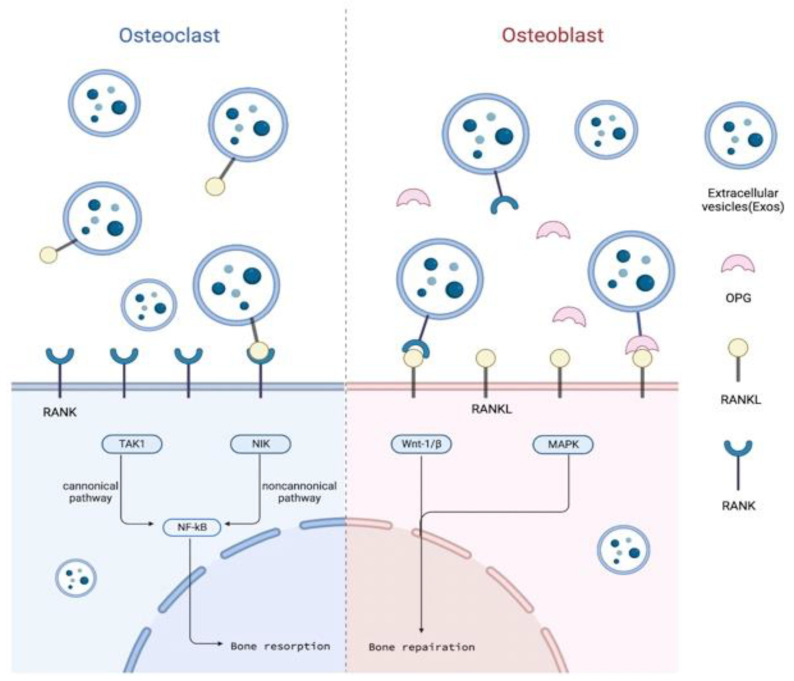
RANKL/RANK/OPG signaling pathway in alveolar bone. SC-EVs can act on osteoblasts and osteoclasts to adjust the balance and affect the reconstruction of alveolar bone through the RANKL/RANK/OPG pathway. (This figure was drawn using BioRender.com).

In the intricate signaling pathway network of bone metabolism, the RANKL/RANK/OPG signaling pathway is a key link. This signaling pathway participates in the whole process of alveolar bone metabolism through both the canonical pathways of NF-κB through TRAFs, as well as the non-canonical pathways of NF-κB, respectively, by TRAFs and NIK [60,61,62] (Figure 3). Some relative studies have observed that both RANK and RANKL expression was widely found in the gingival epithelium and the lamina propria, while OPG was mainly located in the connective tissue [63]. In addition, the RANKL/RANK/OPG pathway is vital in periodontitis, and one of the periodontal therapies is to reduce the level of RANKL in the gingival, or rather to decrease the RANKL/OPG ratio. Confirming whether the RANKL/RANK/OPG pathway is involved in the osteogenesis of EVs expression or not, as well as understanding its mechanism, are necessary requirements for SC-EVs to become a clinical therapy.

At the starting of the method of fortify osteoclast separation and actuation, bone–lining cells and other tranquil osteoblast cells obtain signals, and, at that point, they begin to discharge cytokines related to resorb bone, such as RANKL and the macrophage colony-stimulating calculate (M-CSF) [64]. M-CSF and RANKL are parts of extracellular signaling. During osteoclastogenesis, the combination of RANKL and RANK dynamically initiates intracellular signaling, including NF-κB signaling and MAPK signaling [65]. M-CSF stimulates the monocytes to differentiate into osteoclast precursors, and RANKL induces the formation of multinucleated osteoclasts [66]. Many inflammatory mediators are involved in the inflammatory bone resorption induced by periodontitis. When toll-like receptors on the surface of osteocytes recognize lipopolysaccharides on the surface of Gram-negative bacteria, the MAPK/ERK signaling pathway is stimulated, resulting in the upregulation of interleukin-6 (IL-6) expression [67], which triggers the activation of the Janus kinase (JAK). The JAK enters the nucleus by phosphorylating a transcriptional activator (STAT) to upregulate the RANKL expression in bone cells [68]. In addition to cytokines, there are many stimulating factors and signal pathways that regulate the activity of osteoblasts and osteoclasts [69]. Two mechanical forces, tensile stress and compressive stress, are involved in the OTM process. Osteoclasts and osteoblasts are activated on the compressive side and the tensile side, respectively, and they both are differentiated from stressed PDL stem cells [70].

When bone resorption occurs to a certain extent, signal transduction reverses into osteoblasts. Osteoclasts are released from transforming growth factor-β, which could stimulate Wnt1 secretion and osteogenesis. Osteoclasts produce multiple factors to promote bone formation. Developed osteoclasts release exosomes, whose surfaces contain RANK, and then the exosomes combine with osteoblasts, logically activating osteoblasts. OPG released from osteoblasts blocks up excessive bone resorption by integrating self-expressed RANKL [71,72].

This cycle, among formation and resorption, is monitored precisely and builds the reconstruction of alveolar bone.

### 5.1. Outline of RANKL/RANK/OPG Signaling Pathway and Alveolar Bone Osteogenesis

RANK, also known as TNFRSF11, is a member of the tumor necrosis factor receptor family and is a type I trimerized transmembrane protein on osteoclasts. RANKL, also called TNFSF11, is a homomeric type II transmembrane protein on the surface of osteo-blasts. During osteoclastogenesis, RANKL binds to RANK to activate NF-κB translocation into the nucleus, triggering osteoclast gene transcription [73]. As a soluble bait receptor of RANKL, OPG competes with RANK. Thus, it prevents the osteoclast process that is activated by the RANKL/RANK pathway and inhibits bone resorption [74].

The alveolar bone cells in the oral cavity are constantly undergoing cell metabolism, generating bone, or resorbing bone and causing the alveolar bone to undergo physiological and pathological remodeling. Physiological alveolar bone metabolism includes tooth eruptions and OTM.

Castaneda et al. [75] found that the overexpression of RANK in the alveolar bone can increase the amount of TRAP+ cells and improve osteoblast development regulator Runx2, as well as causing an acceleration of early tooth eruptions. Early in tooth and periodontal development, using a RANKL-blocking antibody could temporarily inhibit bone resorption and delay tooth eruptions [76]. It was deduced that RANKL/RANK are involved in the bone metabolism of tooth eruptions.

OTM is the process of the selective absorption and deposition of different alveolar bones under the action of force. Studies have found that, during OTM, the expression of RANK increases, and there is a certain link between RANK and osteoclastogenesis [77]. In another experiment, a low-energy laser was used to irradiate alveolar bone to stimulate osteogenesis and to speed up the movement of teeth. The research found that RANKL/RANK increased significantly in a targeted method, while OPG did not increase significantly [78].

In pathological alveolar bone metabolism, periodontitis is the main manifestation. Studies have found that WP9QY, a RANKL-binding peptide, can block RANK-induced osteoclasts, and WP9QY may be an effective drug for preventing bone loss in periodontitis. Researchers have used WP9QY to treat OPG-deficient mice with periodontitis, and micro-CT images confirmed that the interdental bone of the mandibular first molar had obvious osteogenesis and an increased bone density [79]. In an inflammatory environment, Th17 cells in periodontal connective tissue produce IL-17, which stimulates the combining of RANKL and RANK, leading to bone loss [80].

Through the above experimental results and research conclusions, it can be seen that the RANKL/RANK/OPG is involved in alveolar bone metabolism. The expression site, expression content, and inhibitors of RANKL/RANK/OPG can affect bone formation and resorption. These encourage us to still further figure out the osteogenic role of the RANKL/RANK/OPG pathway in SC-EVs.

### 5.2. SC-EVs in Alveolar Bone Osteogenesis through RANKL/RANK/OPG Pathway

The increased levels of RANKL in crevicular fluid or a high RANKL/OPG ratio often indicated osteopenia, a risk of alveolar bone resorption, or periodontitis [81,82,83]. Reducing the expression of RANKL or adjusting the propotion of RANKL/OPG is an effective strategy for preventing alveolar bone resorption and assisting osteogenesis. In the human body, osteocytes or osteoblasts tend to be spatially distant from osteoclasts, and RANKL tends to be distant from RANK, as well [84]. Tissue-engineering treatments implant stem cells or SC-EVs combined with active molecules/scaffolds into the bone defect area, which can directly promote the RANKL/RANK/OPG signaling pathway, locally, induce osteogenic differentiation, and enhance the formation of new bone [85].

Liu et al. [86] discovered periodontal osteogenic regeneration that was caused by BMSC-sEVs, which is favorable evidence for a cell-free therapy for periodontal regeneration and also affirms the effect of the RANKL/RANK/OPG pathway. They loaded the BMSC-sEVs into a hydrogel and then injected it into experimental periodontitis rats to verify the therapeutic effects. At four to eight weeks after administration, micro-CT illustrated that the alveolar bone misfortune within the BMSC-sEVs-hydrogel bunch were less than the control gather. Histological examination showed that aggravation and collagen destruction were, moreover, decreased. Encouragingly enough, the proportion of RANKL/OPG went down, and TGF-β1 and the extent of M2/M1 macrophage expanded when infused with BMSC-sEVs. From the examination, it was hypothesized that BMSC-sEVs advanced the recovery of periodontal tissue. BMSC-sEVs may intervene in the work of osteoclasts through the RANKL/RANK/OPG pathway to repress periodontal tissue harm, and they may influence macrophage polarization to control the incendiary safe reaction.

ADSCs are rich in the human body and easily extracted; they are a common source cells of SC-EVs [87,88]. Lee et al. [89] treated mice with osteoporosis in three groups: one that received an intravenous injection of ADSC-EVs (1 × 108 or 5 × 108 particles/100 μL PBS), one that received an intravenous injection of OPG-depleted ADSC-EVs (1 × 108 or 5 × 108 particles/100 μL PBS), and a control group. Bone loss was essentially diminished in ADSC-EV gathering, and BMSCs movement was advanced. In any case, the gather infused with OPG-depleted ADSC-EVs did not appear as an inclination towards a lessening in bone resorption, showing that OPG is imperative for the treatment impact of ADSC-EVs. As a non-cellular treatment for treating bone loss and assisting in osteogenesis, the restorative impact of SC-EVs depends to a certain degree on the substance of OPG.

Dental stem cells, such as GMSCs [90], apical papilla stem cells (SCAP) [91], and dental follicle stem cells (DFSCs) [92], are often used in oral stem cell therapy [93]. The advantages of using dental stem cells include the release of a large amount of EVs [93], immune regulation [94], and the ability to perform minimally invasive extractions to reduce patient discomfort [95].

Lipopolysaccharides (LPSs) are components of the outer membrane of Gram-negative bacteria, are components of the outer cell membrane of Gram-negative bacteria, and are one of the main inducements of periodontal injury. However, the damage to soft and hard tissues directly caused by LPSs is limited; they are more used as a bacterial virulence component to activate the host’s immune inflammatory reaction. Studies have found that the inflammatory microenvironment induced by LPS can enhance the proliferation, differentiation, and adhesion abilities of dental follicle cell (DFC), which is conducive to periodontal regeneration, and this success could possibly be attributed to exosomes, as well [96]. The study showed that, in experimental periodontitis rats, LPS-preconditioned DFC-derived sEVs (L-DFC-sEVs) were loaded into hydrogel and applied to the treatment of periodontitis in vivo, which could partially reduce the expression of the RANKL/OPG. It is beneficial to repair the missing alveolar bone in the early stages of treatment, as well as preserve alveolar bone in the later period. Regrettably, the main components of L-DFC-sEVs and the underlying mechanism of osteogenesis were not explained by the experiment.

Based on this experimental plan, Huang et al. [97] performed a proteomic analysis on the components of L-DFC-sEVs and applied them to a canine periodontitis model. The results confirmed that, under inflammatory conditions, L-DFC-sEVs might inhibit the ROS/JNK signal pathway to decrease the proportion of PDLSCs RANKL/OPG and to promote the M2 polarization through ROS/ERK signal pathway, ultimately restraining alveolar bone loss and promoting osteogenesis. The above two experiments illustrate that the osteogenesis of SC-EVs involves the RANKL/RANK/OPG pathway, and at the same time, they emphasize that stem cells can still secrete EVs in an inflammatory environment to regulate the local inflammatory microenvironment. The collected SC-EVs had stronger antioxidant and anti-inflammatory effects than those collected from normal tissues.

## 6. Conclusions

In summary, many studies have shown that SC-EVs obtained from various tissues and combined with bioactive substances, such as hydrogels or scaffolds, can effectively promote osteogenesis and repair bone defects when used as bone tissue engineering technology. The possibility of using SC-EVs as a cell-free therapy is now a popular area of medical research. More and more studies have shown that the use of SC-EVs for alveolar bone osteogenesis is safe and effective, and it is expected to provide new ideas for oral clinical treatments. However, there are still some problems to be solved, such as the selection of the best source of stem cells, the selection of the appropriate diameter of EVs, the best transplantation method, the optimal number of EVs, the selection of new materials, and finally, which patients are suitable for treatment with SC-EVs, etc. Although there are still many problems to be solved, we already know that SC-EVs and the RANKL/RANK/OPG pathway have a significant connection in periodontal osteogenesis. They may represent an effective means to help clinicians solve problems, such as an insufficient periodontal bone mass, orthodontic tooth movement, or even inflammatory alveolar bone loss, such as that caused by periodontitis.

## Data Availability

Data availability is not applicable to this article as no new data were created or analyzed in this study.
